# Efficacy and safety of hydromorphone for preemptive analgesia in patients undergoing laparoscopic cholecystectomy: a systematic review and meta-analysis

**DOI:** 10.1186/s12871-025-03420-4

**Published:** 2025-11-17

**Authors:** Rongrong Li, Hao Yin, Weijun Luo, Yutong Yang, Hongbin Yuan, Xingying He

**Affiliations:** Department of Anaesthesiology, Second Affiliated Hospital of Naval Medical University, Shanghai, China

**Keywords:** Hydromorphone, Efficacy, Safety, Meta-analysis

## Abstract

**Background:**

Laparoscopic cholecystectomy (LC) is a widely adopted minimally invasive procedure for the treatment of gallbladder diseases. However, postoperative pain remains a significant clinical challenge, with more than 30% of patients experiencing moderate to severe pain, which may prolong hospital stays and impede enhanced recovery after surgery (ERAS) protocols. Hydromorphone, a potent semisynthetic opioid with rapid onset and prolonged analgesic effects, has been employed for preemptive analgesia in LC. This agent has greater analgesic potency and a potentially more favourable side effect profile than morphine does. This systematic review and meta-analysis aimed to evaluate the efficacy and safety of hydromorphone as a preemptive analgesic in patients undergoing LC.

**Methods:**

A comprehensive literature search was conducted across multiple databases, including PubMed, Cochrane, Embase, Web of Science, CNKI, VIP, Wanfang Data, and CBM, from their inception to 2025. Studies assessing the efficacy and safety of hydromorphone for pre-emptive analgesia in LC recipients were included. Additional relevant publications were identified by reviewing references from retrieved articles and searching for commercial system names. Meta-analyses were performed using fixed- or random-effects models on the basis of heterogeneity levels.

**Results:**

Seventeen studies involving a total of 1,515 patients (hydromorphone group, *n* = 761; control group, *n* = 754) were included. The control group received either normal saline or no treatment. Compared with controls, patients treated with hydromorphone presented significantly decreased pain intensity at multiple postoperative time points (*P* < 0.05); shortened extubation time (*P* < 0.05); a reduced incidence of emergence agitation (*P* < 0.05); and improved haemodynamic stability, as evidenced by reduced numbers of fluctuations in heart rate and mean arterial pressure (*P* < 0.05). Additionally, hydromorphone was associated with decreased levels of inflammatory markers (IL-1, IL-8, and TNF-α; *P* < 0.05). No significant difference was observed in the incidence of postoperative nausea and vomiting (PONV) between the two groups.

**Conclusion:**

The outcomes of this study substantiate the efficacy and safety of hydromorphone in the management of postoperative pain, demonstrating superiority over placebo or no treatment.

## Introduction

In recent years, laparoscopic cholecystectomy (LC) has gained widespread clinical adoption owing to its minimally invasive nature, rapid postoperative recovery, and technical feasibility [1]. However, despite these advantages, the surgical procedure—which includes pneumoperitoneum establishment, abdominal wall trocar insertion, organ manipulation, and electrocautery—frequently induces significant postoperative pain. Current evidence indicates that more than 30% of LC recipients experience moderate to severe postoperative pain [2], which can trigger substantial physiological stress responses and potentially compromise recovery outcomes. The selection of appropriate analgesic agents is therefore crucial for ensuring surgical success, maintaining haemodynamic stability, facilitating prompt recovery from anaesthesia, and minimizing postoperative complications. Hydromorphone, a semisynthetic opioid agonist with high µ-receptor affinity, has emerged as an attractive option for perioperative pain management. Its distinct pharmacological profile, characterized by rapid onset (attributed to enhanced lipophilicity) and a potent analgesic effect (approximately five times that of morphine), makes it particularly effective for acute postoperative pain control [3]. Furthermore, its balanced hydrophilic and lipophilic properties contribute to sustained analgesia while potentially reducing adverse effects.

Given the growing body of clinical research evaluating the application of hydromorphone in LC, we conducted this systematic review and meta-analysis to comprehensively assess its efficacy and safety profile in this surgical context.

## Methods

### Search strategy

The data collection and analysis were conducted in accordance with the Cochrane Collaboration guidelines for best practices and the Preferred Reporting Items for Systematic Reviews and Meta-Analyses (PRISMA) guidelines [4]. The protocol for this meta-analysis is available in PROSPERO (CDR42024520173). English- and Chinese-language literature databases, including PubMed, the Cochrane Library, Embase, Web of Science, CNKI, Wanfang, VIP and CBM, were electronically searched from inception to February 2025 to collect eligible randomized controlled trials (RCTs). Additionally, a trace was conducted on the included literature, which was limited to Chinese- or English-language publications. Searches were performed according to the retrieval rules of the respective databases, using a combination of subject terms and free-text words. The search terms included “hydromorphone”, “cholecystectomy”, “randomized controlled trial”, and “RCT”. The general search strategy for PubMed is given in Table 1.

**Table 1:** Search strategy

((Hydromorphone[Title/Abstract] OR Dilaudid[Title/Abstract]) AND (Cholecystectomy[Title/Abstract] OR Laparoscopic[Title/Abstract] OR LC[Title/Abstract]) AND "Randomized Controlled Trial"[pt])	

### Inclusion and exclusion criteria

The inclusion criteria were as follows: (1) Study population: patients with American Society of Anesthesiologists (ASA) physical status classification I to III who underwent laparoscopic cholecystectomy under general anaesthesia, with no restrictions on age or sex. (2) Study type: randomized controlled trial. (3) Intervention: the experimental group received intravenous administration of hydromorphone, whereas the control group received a placebo or no treatment. (4) Primary outcome measures: postoperative visual analogue scale (VAS) scores at different time points. Secondary outcome measures: ① haemodynamic parameters at different time points during surgery; ② time to extubation; ③ agitation scores during the awakening period; ④ incidence of adverse reactions; ⑤ serum inflammatory markers.

The exclusion criteria were as follows: (1) Animal trials, reviews, abstracts of scientific meetings, unpublished observations, and nonresearch works. (2) Master’s and doctoral dissertations. (3) Literature not written in Chinese or English. (4) Studies with only abstracts and no full text or with full text that did not provide sufficient raw data.

Two reviewers independently screened the literature, extracted data and selected studies for inclusion in the review. Any disagreements were referred to a third reviewer to resolve them and determine whether to extract the data.

### Data extraction and quality assessment

All studies comparing hydromorphone to placebo or no treatment for laparoscopic cholecystectomy were included. Two researchers independently conducted the data screening and extraction. First, duplicate studies were identified and excluded using EndNote software. An initial manual screening was subsequently performed by reading the titles and abstracts. If the titles and abstracts were insufficient for judgement, the full texts were downloaded and read. In cases of disagreement, a third researcher was consulted to reach a consensus. The extracted information included VAS scores, haemodynamic fluctuations, levels of inflammatory factors, stress response, quality of recovery, and adverse reactions.

Two reviewers independently evaluated the risk of bias for each publication using the Cochrane risk of bias tool. Each risk assessment assigned a category of “high”, “low”, or “unclear”. Review Manager (RevMan 5.4) was used to perform the quality assessment.

### Statistical analysis

RevMan 5.4 software was used for the statistical analysis, with the criterion for a significant difference set at *P* < 0.05. For continuous outcomes such as postoperative VAS scores and haemodynamic parameters, the Mantel‒Haenszel chi-square test was used, and the results are expressed as the mean difference with a 95% confidence interval (CI). For dichotomous data, the inverse variance method was used, and the results are expressed as risk ratios with 95% CIs. In both cases, *P* < 0.05 was considered the criterion for a statistically significant difference.

Heterogeneity was assessed using the I² statistic. If I²≤50% and *P* ≥ 0.1, the heterogeneity among studies was considered acceptable, and a fixed-effects model (FEM) was used for the meta-analysis. If I²>50% and *P* < 0.1, significant heterogeneity was inferred to be present among the studies, and a random-effects model (REM) was used for the meta-analysis. If there was considerable heterogeneity among studies, sensitivity analysis was conducted to identify the source of heterogeneity and minimize it as much as possible. The z test was used to analyse the pooled statistics, and publication bias was visualized using funnel plots.

## Results

### Study selection

**Fig. 1:**
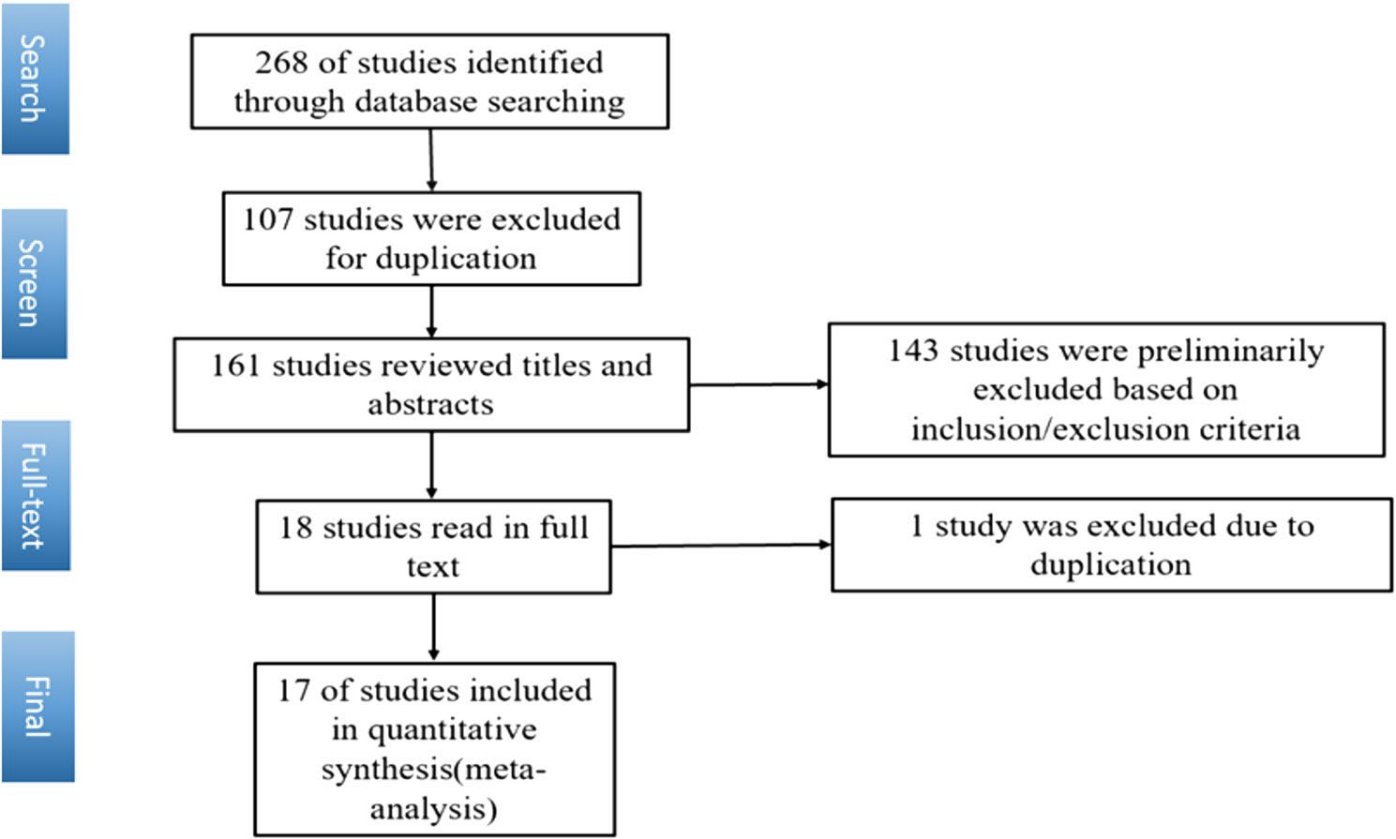
Flow chart of literature search process

A total of 268 relevant studies were included according to the search strategy after duplicates were removed by using EndNote, and the full text of each identified study was screened. Ultimately, 17 RCTs were selected for meta-analysis (Fig. 1) [5–21].

### Study characteristics

Among the 17 included studies, a total of 1,515 patients were included, with 761 patients in the hydromorphone group and 754 patients in the control group. The basic characteristics of the included studies are shown in Table 2.

**Table 2:**
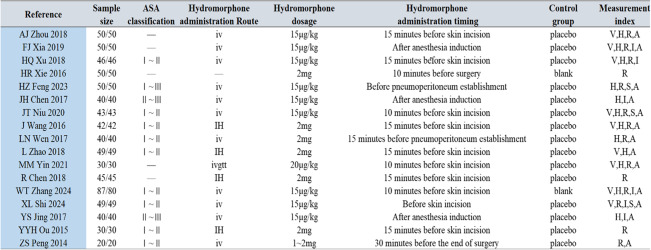
Study characteristics and summary of hydromorphone administration [5–21]

Note on Hydromorphone Administration: The 'Hydromorphone administration Route' column specifies the method by which hydromorphone was administered in the hydromorphone group of each study. The dosage of hydromorphone for each study is detailed in the 'Hydromorphone dosage' column. The 'Hydromorphone administration timing' column indicates when the hydromorphone was administered relative to the surgical procedure

### Study quality and risk of bias

**Fig. 2:**
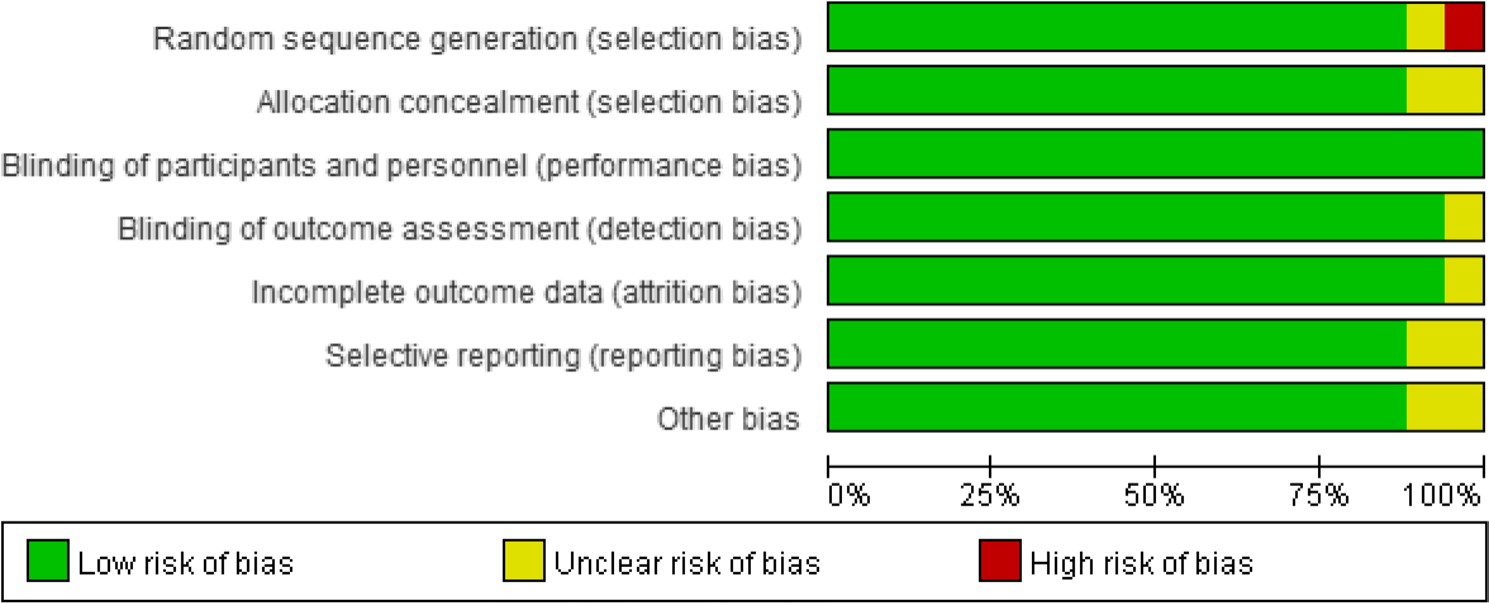
Risk of bias summary plot

**Fig. 3:**
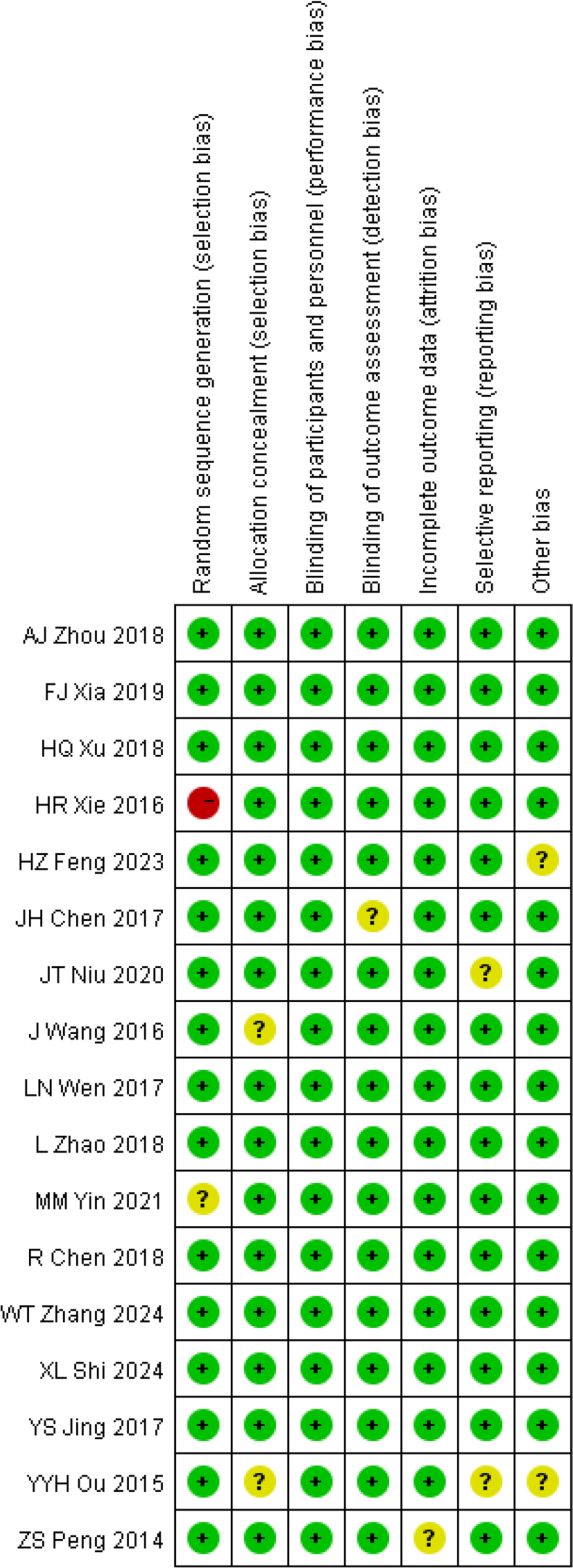
Risk of bias traffic light plot

The risk-of-bias assessment results of the 17 RCTs are presented in Fig. 2. No issues were found regarding incomplete outcome data (attrition bias) in any of the studies. However, in the areas of allocation concealment, blinding of outcome assessment, selective reporting, and other biases, the studies had an unclear risk of bias. Consequently, these factors may have led to bias in assessing adherence to the intervention, causing “some concerns” about many of the studies included in this meta-analysis (Figs. 2 and 3).

### VAS score

**Fig. 4:**
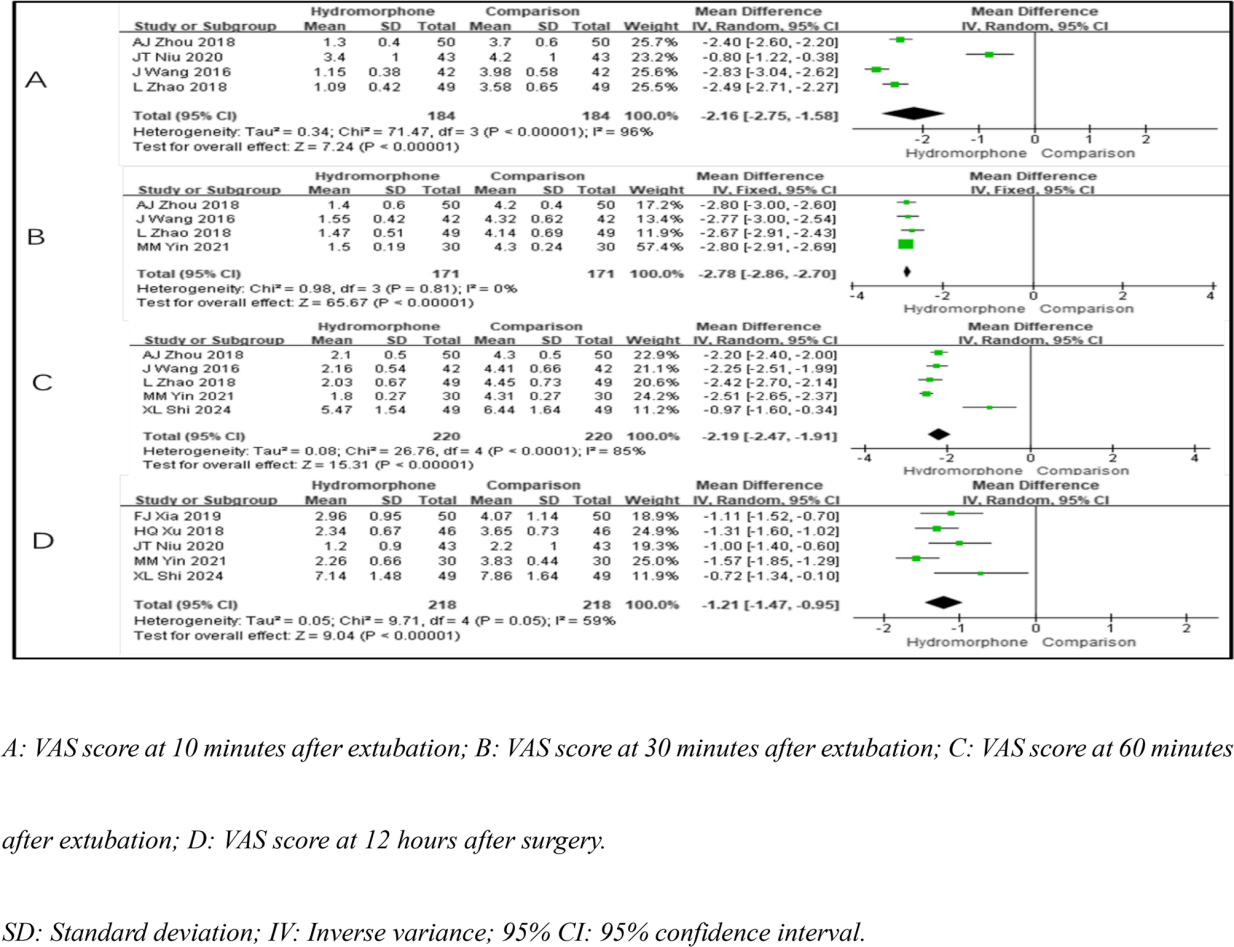
Meta-analysis results of pain intensity of hydromorphone versus placebo

Eight studies [5–7, 10, 12]– [13, 15, 17] reported VAS scores at different time points after extubation: 10 min, 30 min, 60 min, and 12 h postoperatively. The pain scores at 10 min after extubation were significantly heterogeneous (*P* < 0.001, I²=96%), as were the scores at 60 min (*P* < 0.001, I²=85%). The pain scores at 30 min after extubation showed no significant heterogeneity (*P* = 0.81; I²=0%), but there was a moderate level of heterogeneity at 12 h postoperatively (*P* = 0.05; I²=59%). A fixed-effects model was used for the pain scores at 30 min after extubation, whereas a random-effects model was applied to the other time points because of the presence of heterogeneity. The results indicated that compared with the control group, the hydromorphone group had significantly lower pain scores at all time points (the weighted mean difference [MD] was MD10=−2.16, 95% CI [−2.75, 1.58], *P* < 0.05; MD30=−2.78, 95% CI [−2.86, 2.70], *P* < 0.05; MD60=−2.19, 95% CI [−2.47, 1.91], *P* < 0.05; MD12=−1.21, 95% CI [−1.47, 0.95], *P* < 0.05) (Table 3; Fig. 4).

**Table 3:** Meta-analysis of postoperative VAS scores at specified time points

VAS Score	Reference	Heterogeneity			Effect Size	*P*
		*P*	I²(%)	Effect Modle	(MD, 95%CI)	
10 min after extubation	4	< 0.001	96	Random effect	−2.16[−2.75, −1.58]	< 0.001
30 min after extubation	4	0.81	0	Fixed effect	−2.78[−2.86, −2.70]	< 0.001
60 min after extubation	5	< 0.001	85	Random effect	−2.19[−2.47, −1.91]	< 0.001
12 h after surgery	5	0.05	59	Random effect	−1.21[−1.47, −0.95]	< 0.001

Annotation: Reference: The citation of the study. Heterogeneity Analysis: Indicates whether the studies’ results are consistent (I² < 50% and P ≥ 0.1) or show significant heterogeneity (I² >50% and P < 0.1). Effect Size (MD): A negative value suggests lower levels of inflammatory markers in the hydromorphone group compared to the control group

*P*-value: A value less than 0.05 is considered statistically significant, indicating a meaningful difference between groups

### Recovery quality

#### Autonomous breathing recovery time

**Fig. 5:**
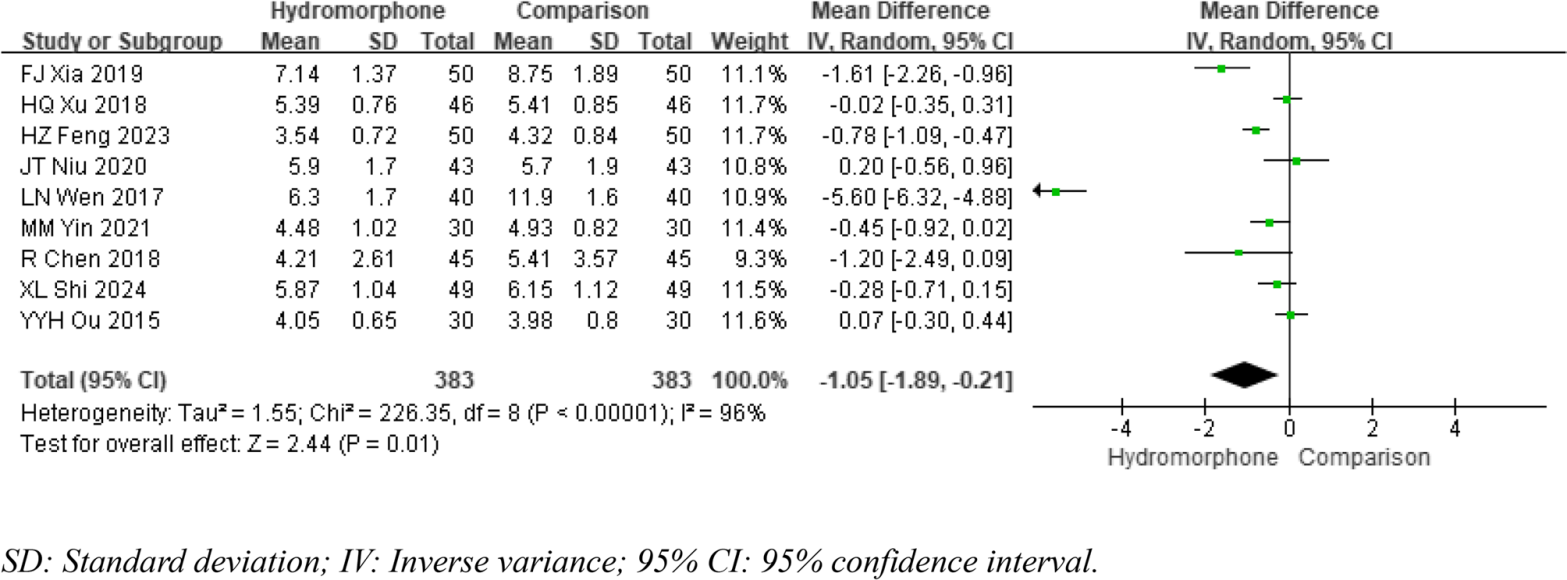
Meta-analysis results of autonomous breathing recovery time of hydromorphone versus placebo

Nine studies [6]– [7, 9, 12, 14–17, 19] provided data on the time to spontaneous respiratory recovery after surgery, and significant statistical heterogeneity was noted among the studies (*P* < 0.001, I²=96%). A random-effects model was used to analyse the data. The meta-analysis revealed that, compared with the control group, the hydromorphone group had a significantly shorter time to spontaneous respiratory recovery (MDA=−1.05, 95% CI [−1.89, −0.21], *P* < 0.05) (Fig. 5).

#### Extubation time

**Fig. 6:**
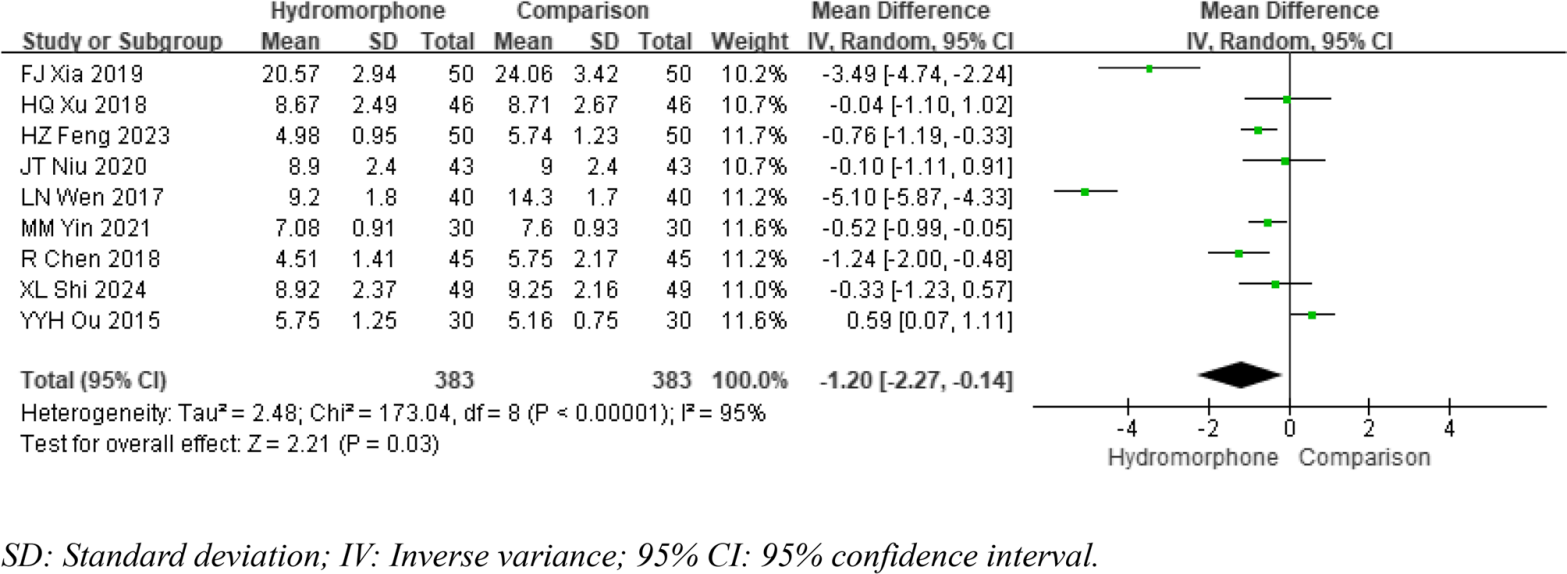
Meta-analysis results of extubation time of hydromorphone versus placebo

Nine studies [6]– [7, 9, 12, 14–17, 19] reported the time to postoperative extubation, and there was significant statistical heterogeneity among these studies (*P* < 0.001, I²=95%). A random-effects model was utilized for data analysis. The meta-analysis demonstrated that compared with the control group, the hydromorphone group had a significantly shorter extubation time (MDE=−1.20, 95% CI [−2.27, −0.14], *P* < 0.05) (Fig. 6).

### Haemodynamic fluctuations

#### Heart rate (HR)

**Fig. 7:**
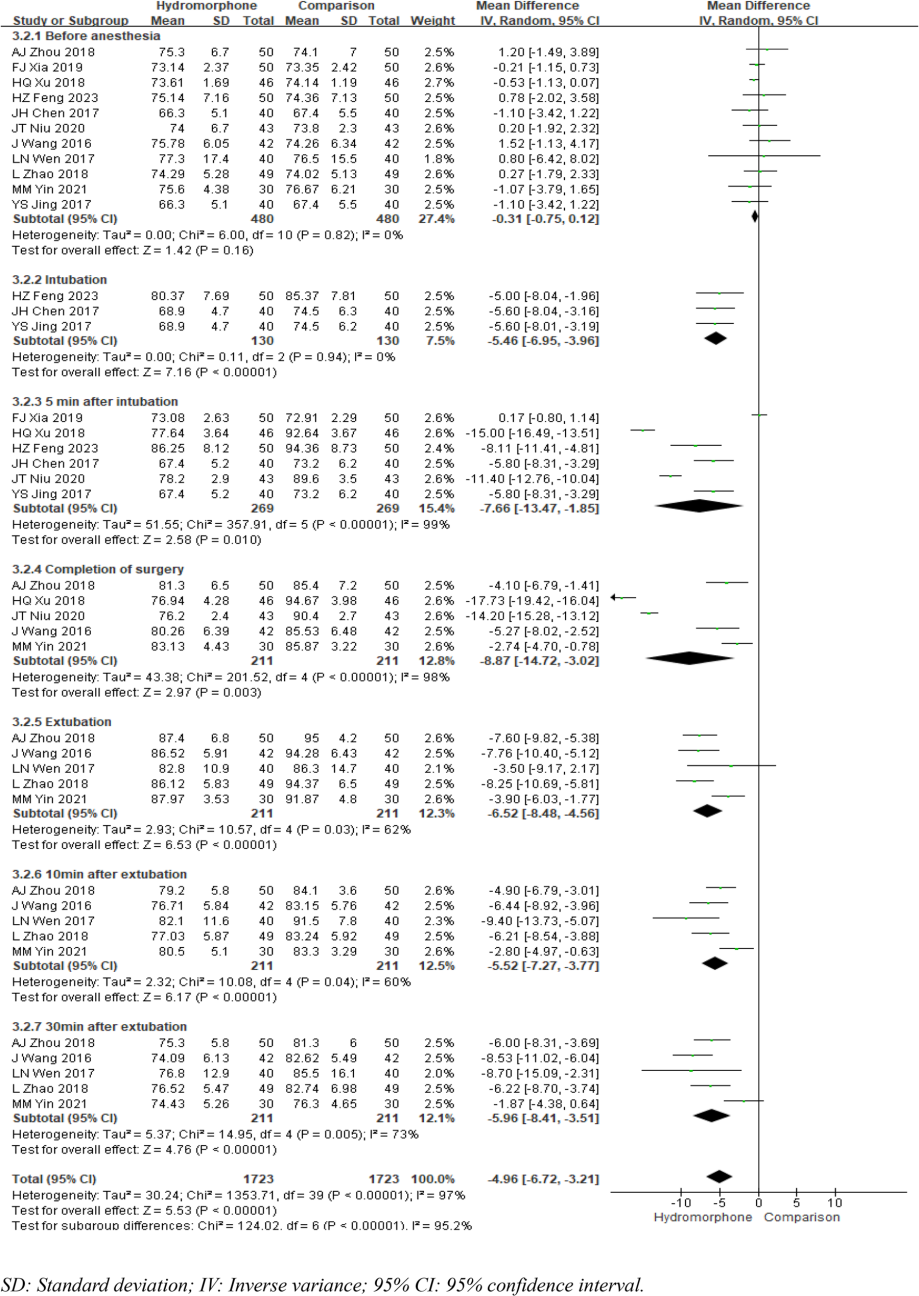
Meta-analysis results of heart rate at different times of hydromorphone versus placebo

Eleven studies [5–7, 9–15, 18] reported changes in heart rate at various time points: before anaesthesia induction, immediately after intubation, 5 min after intubation, at the end of surgery, at extubation, 10 min after extubation, and 30 min after extubation. All these studies compared the baseline heart rate between groups before anaesthesia and included pretreatment data. The heterogeneity analysis revealed *P* = 0.82 and I²= 0, indicating no heterogeneity, and a fixed-effects model was used to analyse the results. The meta-analysis revealed no significant difference in heart rate between the two groups before anaesthesia (MDb=0.31, 95% CI [−0.75, 0.12], *P* = 0.16). Except for the immediate postintubation time point, where no significant heterogeneity was noted, all time points exhibited statistical heterogeneity (*P* < 0.001, I²>50%). A fixed-effects model was used to analyse the heart rate immediately after intubation, whereas a random-effects model was applied to analyse the heart rate at all other time points. The meta-analysis results indicated that compared with the control group, the hydromorphone group had a significantly lower heart rate at all time points. The weighted mean differences (MDs) were MDintubation=−5.46, 95% CI [−6.95, −3.96], *P* < 0.05; MD5min=−7.66, 95% CI [−13.47, −1.85], *P* < 0.05; MDcompletion=−8.87, 95% CI [−14.72, −3.02], *P* < 0.05; MDextubation=−6.52, 95% CI [−8.48, −4.56], *P* < 0.05; MD10min=−5.52, 95% CI [−7.27, −3.77], *P* < 0.05; and MD30min=−5.96, 95% CI [−8.41, −3.51], *P* < 0.05 (Fig. 7).

#### Mean arterial pressure (MAP)

**Fig. 8:**
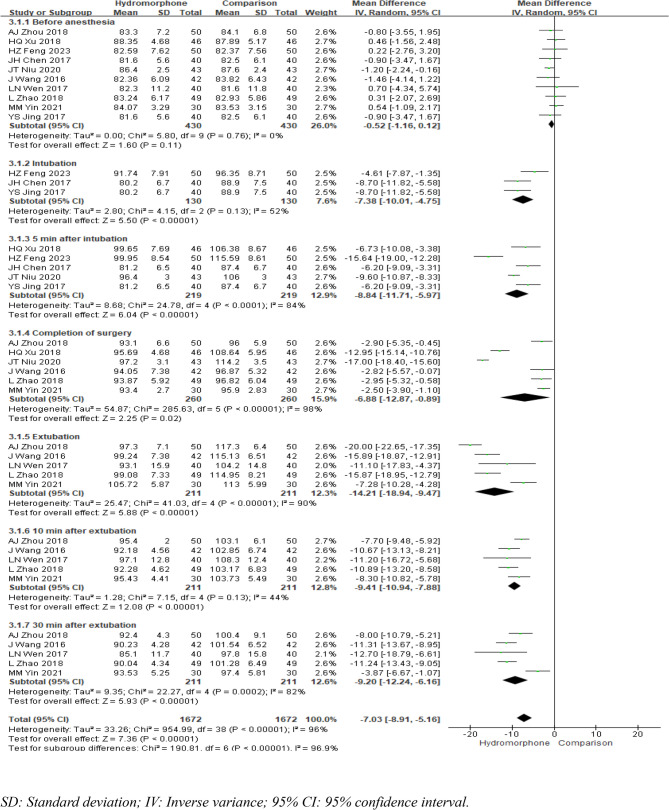
Meta-analysis results of mean arterial pressure at different times of hydromorphone versus placebo

Ten studies [5, 7, 9–15, 18] reported changes in mean arterial pressure (MAP) at various time points: before anaesthesia induction, immediately after intubation, 5 min after intubation, at the end of surgery, at extubation, 10 min after extubation, and 30 min after extubation. The heterogeneity analysis for the mean arterial pressure before anaesthesia induction yielded *P* = 0.82 and I²<50, indicating no heterogeneity. The fixed-effects model revealed no significant difference in the mean arterial pressure between the two groups before anaesthesia (MDb=−0.52, 95% CI [−1.16, 0.12], *P* = 0.11). Except for the time point 10 min after extubation, where there was no significant heterogeneity and a fixed-effects model was therefore used for analysis, the mean arterial pressure at all time points was analysed using a random-effects model because of the presence of heterogeneity. The meta-analysis results indicated that the hydromorphone group had a lower mean arterial pressure than the control group did at all time points, and this difference was statistically significant. The weighted mean differences (MDs) were MDintubation=−7.38, 95% CI [−10.01, −4.75], *P* < 0.05; MD5min=−8.84, 95% CI [−11.71, −5.97], *P* < 0.05; MDcompletion=−6.88, 95% CI [−12.87, −0.89], *P* < 0.05; MDextubation=−14.21, 95% CI [−18.94, −9.47], *P* < 0.05; MD10min=−9.41, 95% CI [−10.94, −7.88], *P* < 0.05; and MD30min=−9.20, 95% CI [−12.24, −6.16], *P* < 0.05 (Fig. 8).

### Inflammatory cytokines

**Fig. 9:**
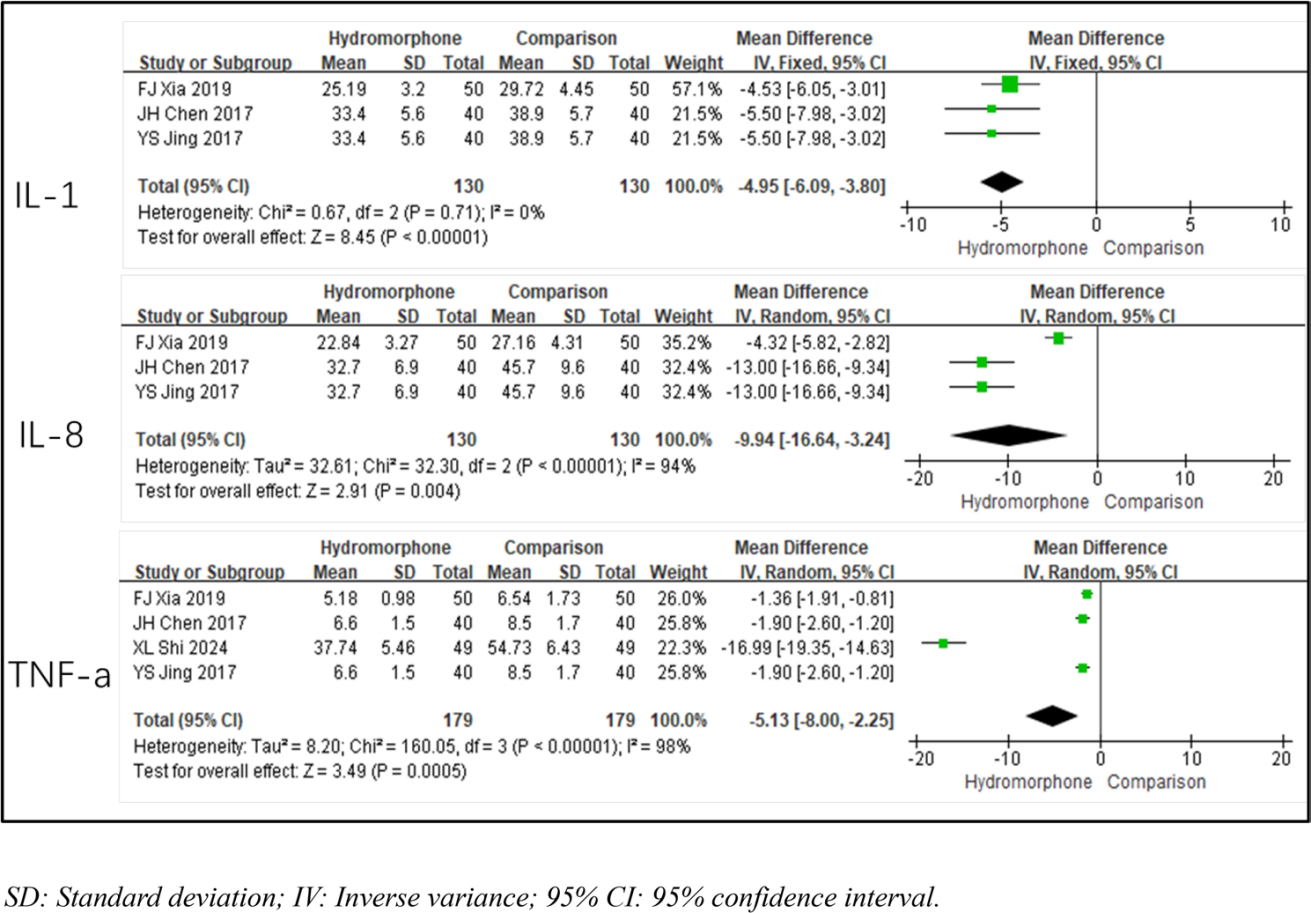
Meta-analysis results of stress response intensity of hydromorphone versus placebo

Four studies [6, 11, 17, 18] reported inflammatory cytokine levels (IL-1, IL-8, and TNF-α) to reflect the degree of stress response in patients. The heterogeneity analysis for IL-1 indicated no significant heterogeneity (*P* = 0.71, I2 < 50), and a fixed-effects model was used for analysis. For IL-8 and TNF-α levels, the heterogeneity analysis revealed significant differences (*P* < 0.01, I2 >50%), and a random-effects model was applied. The results of the meta-analysis suggested that compared with the control group, the hydromorphone group had significantly lower levels of inflammatory markers. The weighted mean differences (MDs) were MDIL-1=−4.95, 95% CI [−6.09, −3.80], *P* < 0.05; MDIL-8=−2.78, 95% CI [−2.86, −2.70], *P* < 0.05; and MDTNF-a=−5.13, 95% CI [−8.00, −2.25], *P* < 0.05) (Table 4; Fig. 9).

**Table 4:** Meta-analysis results of stress response intensity of hydromorphone versus placebo

Inflammatory Factor	Reference	Heterogeneity			Effect Size	*P*
		*P*	I²(%)	Effect Modle	(MD, 95%CI)	
IL-1	3	0.71	0	Fixed effect	−4.95[−6.09, −3.80]	< 0.0001
IL-8	3	< 0.001	94	Random effect	−2.78[−2.86, −2.70]	0.004
TNF-a	4	< 0.001	98	Random effect	−5.13[−8.00, −2.25]	0.0005

Annotations: Displays the differences in stress response intensity for various inflammatory factors (IL-1, IL-6, IL-8, TNF-a) between hydromorphone and placebo groups, including heterogeneity test results and effect sizes

### Adverse events

**Fig. 10:**
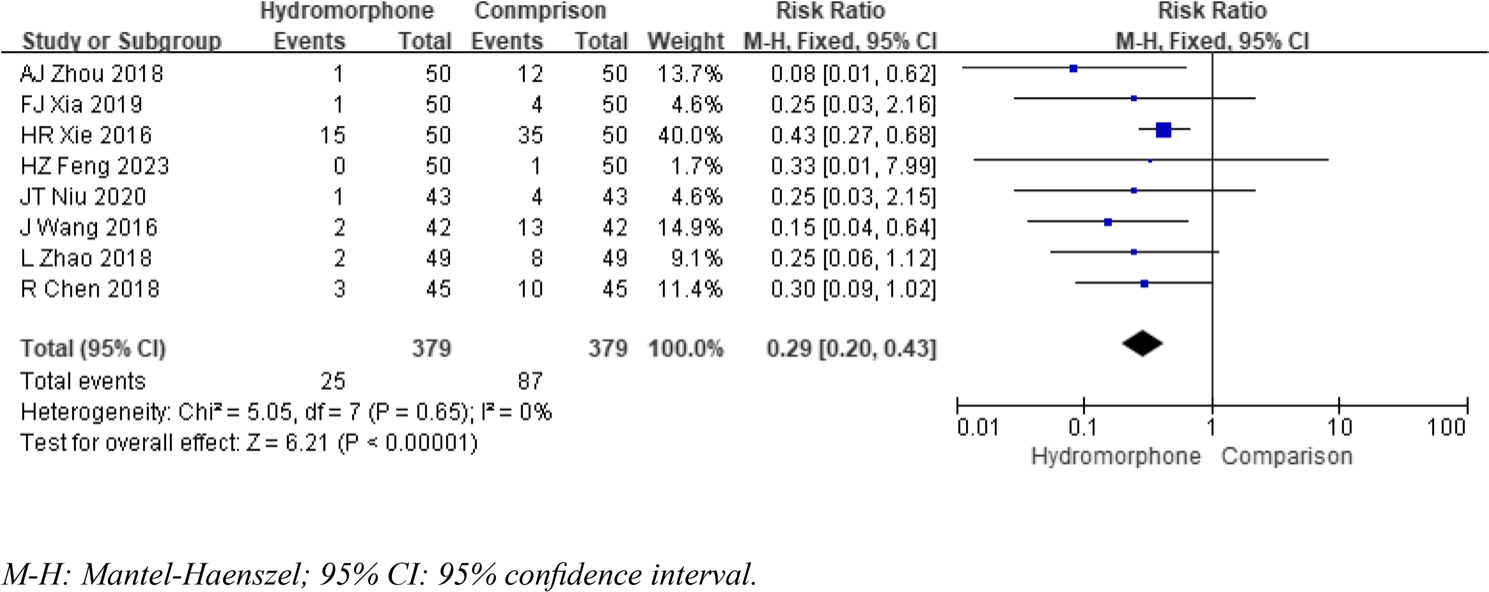
Meta-analysis results of the incidence of restlessness of hydromorphone versus placebo

**Fig. 11:**
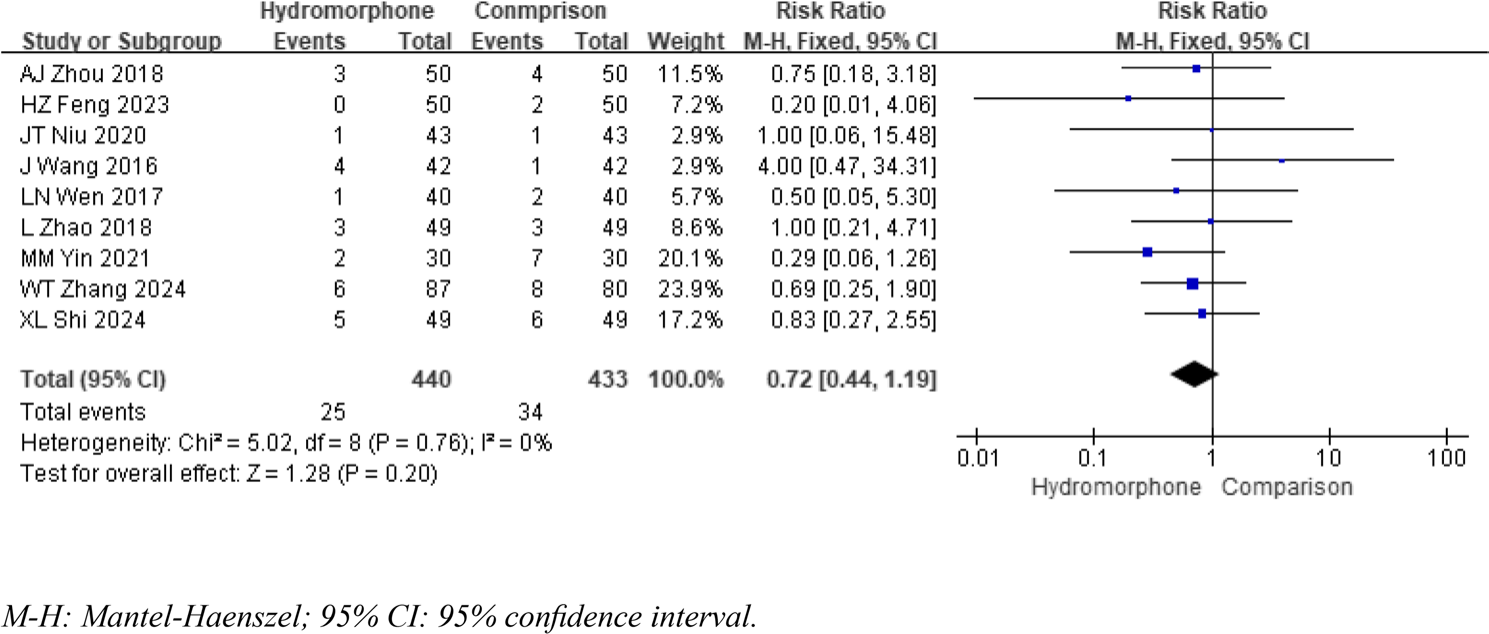
Meta-analysis results of the incidence of nausea and vomiting of hydromorphone versus placebo

Nine studies were included in the statistical analysis of adverse effects, such as emergence agitation [5]– [6, 8–10, 12]– [13, 16] and nausea/vomiting [5, 9]– [10, 12–15, 17, 21]. The heterogeneity analysis (*P* >0.1, I2 < 50%) revealed no significant differences. The meta-analysis results indicated that the incidence of emergence agitation was significantly lower in the hydromorphone group than in the control group. The incidence of nausea/vomiting was also lower in the hydromorphone group, but this difference was not statistically significant. The weighted mean differences (MDs) for emergence agitation and nausea/vomiting, respectively, were as follows: ORR = 0.17, 95% CI [0.10, 0.30], *P* < 0.05; ORNV = 0.71, 95% CI [0.41, 1.20], *P* >0.05 (see Figs. 10 and 11). Other studies reported the occurrence of adverse effects such as itching, blood pressure changes, and respiratory depression. Owing to the limited number of included studies and the lack of significant differences (*P* >0.05), no statistical analysis was conducted.

### Publication bias

**Fig. 12:**
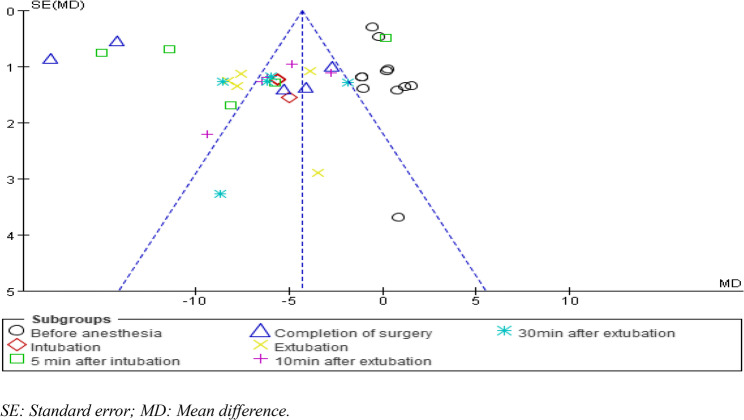
Funnel plot for assessing publication bias of the fluctuation of heart rate at different time points

**Fig. 13:**
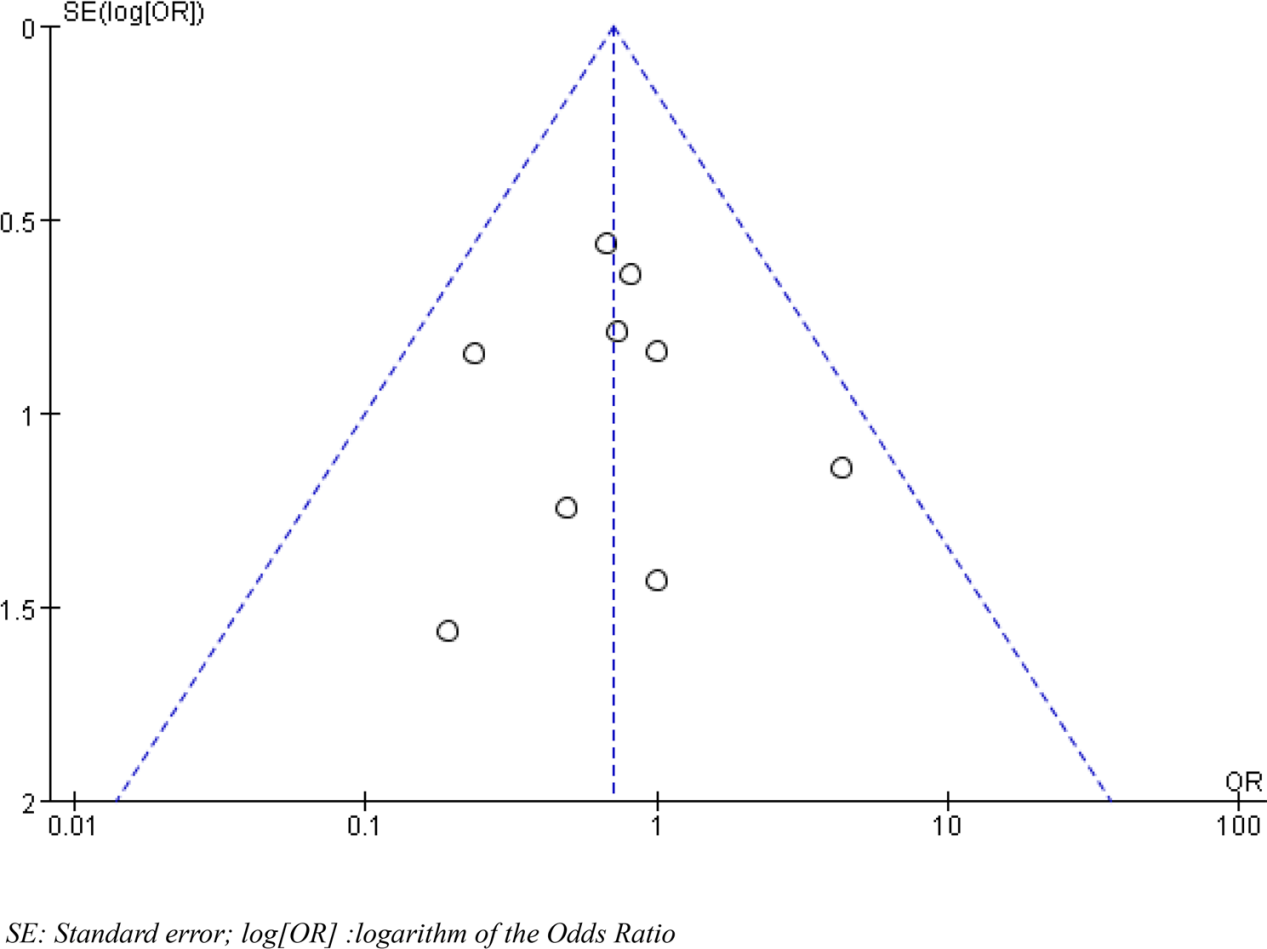
Funnel plot for assessing publication bias of nausea and vomiting

Funnel plots were created to analyse the fluctuations in heart rate at different time points and the incidence of postoperative nausea and vomiting. The distributions on these inverted funnel plots were asymmetrical, suggesting that the included literature may have had significant publication bias and clinical heterogeneity (Figs. 12 and 13).

## Discussion

Since its introduction in the late 1980 s, LC has emerged as the gold standard for minimally invasive gallbladder surgery. The evolution of surgical techniques, including the development of three-port, two-port, and even single-port approaches, has progressively minimized surgical trauma [21]. Despite these technological advancements, clinical evidence indicates that approximately 50% of patients may develop persistent or intermittent upper abdominal pain after surgery [5]. Pain can trigger a stress response, including perioperative haemodynamic fluctuations, postoperative delirium, and enhanced inflammatory responses [22], which may interfere with ERAS. Accordingly, the optimization of perioperative pain management has emerged as a critical focus in contemporary surgical practice. This clinical priority has evolved in parallel with significant paradigm shifts in perioperative care, particularly through the implementation of enhanced recovery after surgery. These evidence-based protocols emphasize multimodal analgesic approaches, regional anaesthesia techniques, and judicious opioid utilization to avoid adverse opioid-related effects [23, 24].

However, opioid receptor agonists are essential components of pain management. Among them, hydromorphone is a potent opioid analgesic. This derivative of morphine is characterized by rapid onset, strong analgesic effects, and mild side effects. Hydromorphone can activate relevant receptors in the patient’s nervous system, block stress signal transduction, and exert analgesic and sedative effects [25]. A volunteer study in the United States [26] showed that compared with morphine, hydromorphone might have greater clinical advantages. At the studied doses, hydromorphone had a rapid onset and high analgesic potency. These findings were further corroborated by Fan et al. [27] in the context of laparoscopic cholecystectomy, where hydromorphone administration not only provided effective postoperative pain relief but also demonstrated immunomodulatory effects through the rebalancing of pro- and anti-inflammatory cytokine responses, ultimately facilitating enhanced surgical recovery.

The results of this meta-analysis indicated that, compared with the placebo control, the use of hydromorphone during LC effectively improved pain intensity scores at 10 min, 30 min, 60 min, and 12 h after surgery, with all the differences being statistically significant. Although the results suggest that hydromorphone is superior to placebo for controlling postoperative pain, importantly, hydromorphone was compared only with an inactive control (i.e., saline or no treatment) in the included studies rather than with other active opioids. This limits the strength of the conclusions that can be drawn regarding its comparative efficacy in clinical practice. Further studies comparing hydromorphone directly with active drug treatments are needed to better understand its role in clinical practice and to provide more comprehensive guidance for clinicians.

The included studies revealed that patients experienced varying degrees of increases in heart rate and blood pressure perioperatively. However, our results revealed that the degree of haemodynamic fluctuation in the hydromorphone group was significantly lower than that in the control group, likely because hydromorphone provides better control of pain and related negative stimuli.

The recovery time of spontaneous breathing and the time to extubation after surgery are important indicators for assessing the quality of patient recovery after general anaesthesia. The recovery of spontaneous breathing is directly related to whether the patient can successfully have the endotracheal tube removed, and the time to extubation reflects the physiological recovery status and safety of the patient after surgery. The results of our meta-analysis suggested that both the time to recovery of spontaneous breathing and the time to extubation were significantly shorter in the hydromorphone group than in the control group, which suggested that compared with control conditions, hydromorphone had a slight effect on respiratory suppression and thus improved perioperative safety.

Emergence agitation (EA) is a condition characterized by psychomotor agitation, hyperactivity, and sensory disturbances that occur during the emergence phase from general anaesthesia and primarily manifests as excessive physical movements [28]. EA may increase the risk of self-injury in patients, thereby affecting their postoperative recovery. To date, the mechanisms underlying EA have not been fully elucidated. According to clinical studies, the incidence of EA in adults ranges from 2.5% to 19%, with pain stimulation and residual anaesthetic drugs being the main causes [29]. The results of this meta-analysis indicated that the use of hydromorphone significantly reduces the incidence of EA, which is advantageous for improving the quality of patient awakening and reducing perioperative complications.

Postoperative nausea and vomiting (PONV) is a common complication associated with opioid use. Notably, this meta-analysis revealed no significant difference in PONV incidence between the hydromorphone and placebo control groups. These findings suggest that hydromorphone may have a reduced propensity to induce PONV, potentially representing a distinct advantage over other opioids. It is well known that PONV risk is influenced by multiple factors, including the surgical procedure type, patient sex and age, timing, dosage, and the concomitant use of other perioperative medications, as revealed in the included studies (see Table 5 for anaesthetic regimens). Additionally, the timing of hydromorphone administration varied across the included studies, which may have influenced the occurrence of PONV (see Table 2 for details of hydromorphone administration). Future studies should therefore focus on identifying optimal hydromorphone administration strategies to further minimize the risk of PONV.

**Table 5:**
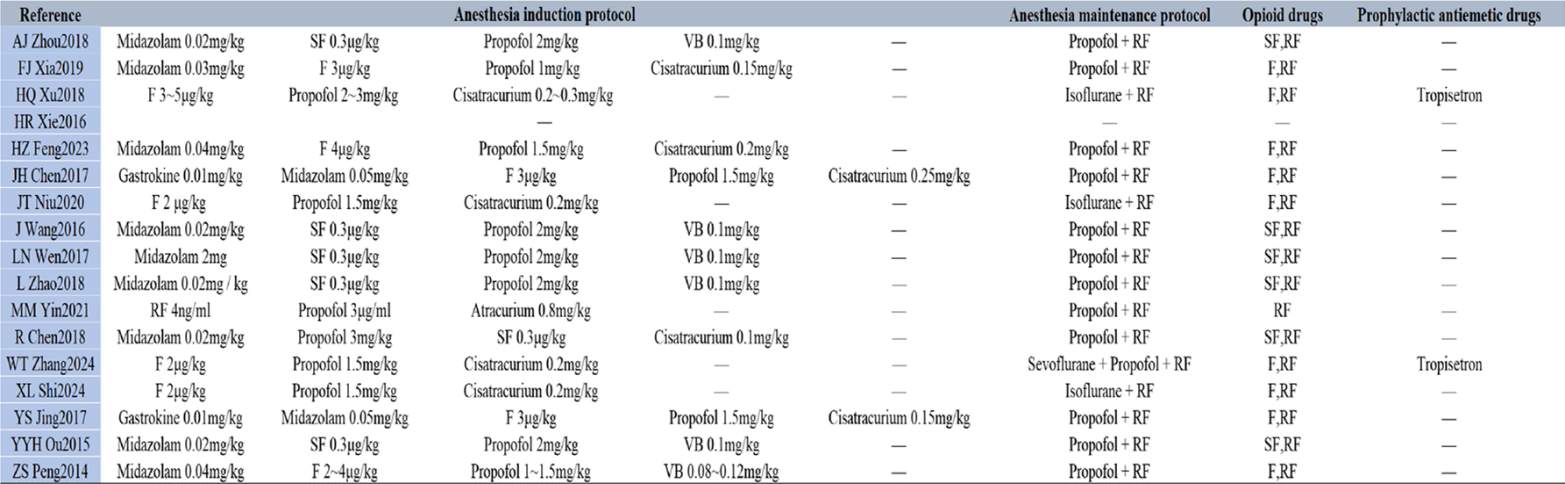
Anesthetic regimens of included studies [5–21]

Annotation: *RF* Remifentanil, *SF* Sufentanil, *F* Fentanyl, *VB* Vecuronium Bromide

The inflammatory response is a natural protective reaction against surgical trauma. However, if the inflammatory response is excessive or dysregulated, it may have a series of harmful effects on patients. IL-1, IL-8, and TNF-α are cytokines that play key roles in the inflammatory response and the progression of various diseases. Members of the IL family play key roles in multiple inflammatory diseases, with a variety of local and systemic effects. IL-1 exerts proinflammatory effects by binding to its corresponding receptors, regulating the transcription levels of its targets, and participating in the processing of precursor enzymes [30]. IL-8 is a major chemokine induces inflammatory responses; its role in the inflammatory response includes participating in the activation of neutrophils, inducing the migration of immune cells to the site of infection, and promoting inflammatory responses [31]. TNF-α is a proinflammatory cytokine that participates in normal inflammatory and immune responses and can synergistically regulate the production of other cytokines, cell survival, and death [32]. The results of this meta-analysis suggested that the levels of postoperative inflammatory cytokines (IL-1, IL-8, and TNF-α) were significantly lower in the hydromorphone group than in the control group, which might be related to rapid completion of ERAS.

## Limitations

The temporal scope and geographic coverage of this meta-analysis present notable limitations. The included studies appear relatively dated, raising concerns about the applicability of their findings to contemporary clinical practice. Advances in surgical techniques, anaesthesia protocols, and perioperative pain management strategies necessitate more current research to evaluate the role of hydromorphone within modern clinical contexts. Furthermore, 16 of the 17 included studies were conducted in China, indicating that the current conclusions primarily reflect Eastern Asian populations. Additional multinational studies involving ethnically diverse cohorts are needed to validate these findings and enhance their external validity.

In addition, variability in anaesthetic protocols (see Table 5 for anaesthetic regimens) and altered expectations and behaviours among participants or researchers were observed in some trials, potentially introducing bias into treatment effect estimates. Although the risk of other forms of bias was generally low, the potential impact of these behavioural factors on the interpretation of the results warrants careful consideration.

## Conclusion

Compared with placebo, hydromorphone administration during LC significantly reduced postoperative pain intensity scores at 10, 30, and 60 min and 12 h after surgery. Additional advantages included improved intraoperative haemodynamic stability, enhanced postoperative recovery quality, and attenuated inflammatory responses. Notably, hydromorphone, in contrast to other opioid analgesics, did not cause more PONV compared with the placebo control. Future research should prioritize the evaluation of hydromorphone within contemporary surgical settings, incorporating multimodal analgesic approaches and patient-centred outcomes.

## Data Availability

All data generated or analyzed during this study are included in this published article and its supplementary information files. The datasets used and/or analyzed during the current study are available from the corresponding author on reasonable request.

## Abbreviations

ASA: American Society of Anesthesiologists
VAS: Visual Analogue Scale
HR: Heart Rate
MAP: Mean Arterial Pressure
IL: Interleukin
TNF-α: Tumor Necrosis Factor-alpha
PONV: Postoperative Nausea and Vomiting
EA: Emergence Agitation
ERAS: Enhanced Recovery After Surgery
ASA: American Society of Anesthesiologists
RF: Remifentanil
SF: Sufentanil
F: Fentanyl
VB: Vecuronium Bromide
PROSPERO: International Prospective Register of Systematic Reviews
